# Phenolic Characterization and Neuroprotective Properties of Grape Pomace Extracts

**DOI:** 10.3390/molecules26206216

**Published:** 2021-10-14

**Authors:** Annalisa Chiavaroli, Marwa Balaha, Alessandra Acquaviva, Claudio Ferrante, Amelia Cataldi, Luigi Menghini, Monica Rapino, Giustino Orlando, Luigi Brunetti, Sheila Leone, Lucia Recinella, Viviana di Giacomo

**Affiliations:** 1 Department of Pharmacy, University G. d’Annunzio, Chieti-Pescara, 66100 Chieti, Italy; annalisa.chiavaroli@unich.it (A.C.); marwa.balaha@unich.it (M.B.); alessandra.acquaviva@unich.it (A.A.); amelia.cataldi@unich.it (A.C.); luigi.menghini@unich.it (L.M.); giustino.orlando@unich.it (G.O.); luigi.brunetti@unich.it (L.B.); sheila.leone@unich.it (S.L.); lucia.recinella@unich.it (L.R.); viviana.digiacomo@unich.it (V.d.G.); 2 Department of Pharmaceutical Chemistry, University of Kafrelsheikh, Kafrelsheikh 33516, Egypt; 3 Genetic Molecular Institute of CNR, Unit of Chieti, University G. d’Annunzio, Via dei Vestini 31, 66100 Chieti, Italy; monica.rapino@unich.it

**Keywords:** neuroprotection, grape pomace, water extract, oxidative stress, BDNF, COX-2, PGE_2_, hypothalamus, catechin(s), *Vitis vinifera*

## Abstract

*Vitis vinifera* (grape) contains various compounds with acknowledged phytochemical and pharmacological properties. Among the different parts of the plant, pomace is of particular interest as a winemaking industry by-product. A characterization of the water extract from grape pomace from Montepulciano d’Abruzzo variety (Villamagna doc) was conducted, and the bioactive phenolic compounds were quantified through HPLC-DAD-MS analysis. HypoE22, a hypothalamic cell line, was challenged with an oxidative stimulus and exposed to different concentrations (1 µg/mL^−1^ mg/mL) of the pomace extract for 24, 48, and 72 h. In the same conditions, cells were exposed to the sole catechin, in a concentration range (5–500 ng/mL) consistent with the catechin level in the extract. Cell proliferation was investigated by MTT assay, dopamine release through HPLC-EC method, PGE_2_ amount by an ELISA kit, and expressions of neurotrophin brain-derived neurotrophic factor (BDNF) and of cyclooxygenase-2 (COX-2) by RT-PCR. The extract reverted the cytotoxicity exerted by the oxidative stimulus at all the experimental times in a dose-dependent manner, whereas the catechin was able to revert the oxidative stress-induced depletion of dopamine 48 h and 72 h after the stimulus. The extract and the catechin were also effective in preventing the downregulation of BDNF and the concomitant upregulation of COX-2 gene expression. In accordance, PGE_2_ release was augmented by the oxidative stress conditions and reverted by the administration of the water extract from grace pomace and catechin, which were equally effective. These results suggest that the neuroprotection induced by the extract could be ascribed, albeit partially, to its catechin content.

## 1. Introduction

Grape (*Vitis vinifera*) worldwide production was estimated to be more than 79 million tons in 2018 according to the Food and Agriculture Organization (FAO—United Nations) [1], and Italy is one of the leading wine-producing countries with numerous varieties of both red and white wines [2]. Every year, millions of tons of residues are generated by the wineries, and there is an urge for waste reduction policy in order to achieve sustainable wine-making processes. Grape pomace is a biodegradable solid by-product of the winemaking process obtained after mechanical press or fermentation and encompassing peels (skin), seeds, and some parts of the stem [3]. Grape pomace contains significant amounts of substances that can be considered beneficial to human health [4]. It mainly consists of dietary fibers that are present in high levels (up to 85% depending upon the grape variety) and polyphenolic compounds that mainly (about 70%) remain in pomace after the winemaking process [5]. Phenolic compounds are secondary plant metabolites which are considered bioactive. It is possible to distinguish an extensive family of phenolic compounds, which are grouped into flavonoids and non-flavonoids. Besides influencing wine quality and properties [6], phenols have potential beneficial effects towards human health. They possess antioxidant, antiviral, antimicrobial, and anti-inflammatory properties which vary with the variety of grapes [7].

There is large evidence of the beneficial role that phenols present in wine regarding how it can impact the nervous system. Antidepressant and anxiolytic properties as well as beneficial effects in Parkinson’s and Alzheimer’s diseases were ascribed to resveratrol, the most studied phenolic compound in wine [8,9]. Additionally, caffeic acid and its natural derivative, caffeic acid phenetyl ester (CAPE), have numerous neuroprotection effects [10] against cancer and others [11]. Other phenols, including catechins, have remarkable neuroprotective activity [12] to such an extent that new therapeutical targets were proposed [8,13].

Many pharmacological activities were ascribed to different parts of grapes [14]. Among others, pomace extract was found effective in positively regulating spermatozoa viability and motility [15], while neuroprotective effects were ascribed to extracts of grape seeds and grapevine leaves [16,17] as well as to grape juice [18]. On the other hand, antioxidant and anti-inflammatory properties of grape pomace extract were investigated [19,20].

With inflammation and oxidative stress at the basis of many neurological disorders, the aim of the present study was the evaluation of the potential neuroprotective activity of a water extract from grape pomace. The Abruzzo region in central Italy is known for its wide variety of red (Montepulciano), rose (Cerasuolo), and white wines. Our research focused on a DOC red wine, the Montepulciano d’Abruzzo variety (Villamagna doc), which was characterized for its content in phenolic compounds. The extract showed protective effects on a hypothalamic cell line exposed to an oxidative stimulus.

## 2. Results and Discussion

### 2.1. Phytochemical Analysis

**Figure 1:**
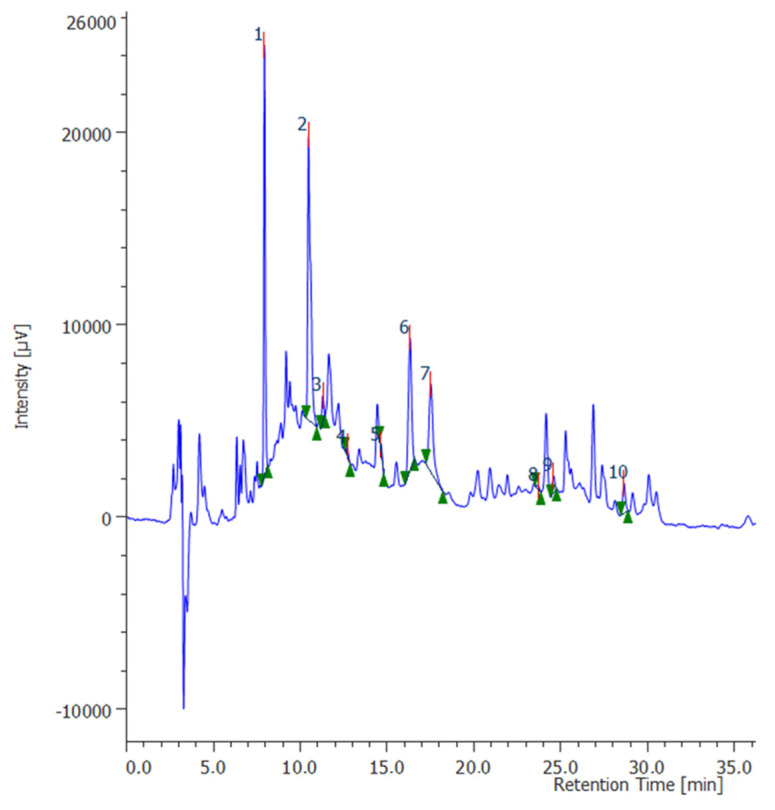
Chromatographic analysis of grape pomace phenolic compounds. The chromatographic analysis confirmed the presence of different phytochemicals: gallic acid (peak #1); caftaric acid (peak #2); catechin (peak #3); chlorogenic acid (peak #4); epicatechin (peak #5); caffeic acid (peak #6); syringic acid (peak #7); coumaric acid (peak #8); ferulic acid (peak # 9).

In the present study, the grape pomace from *V. vinifera* was considered as a high quality by-product for the preparation of bioactive extracts with biological and pharmaceutical interest. In this regard, water was used as elective and biocompatible solvent for such a purpose. The operative extraction conditions were optimized through response surface methodology (RSM). A set of 27 independent runs were performed to evaluate the curvature model, as reported in Table 1. The surface analysis and the analysis of variance (ANOVA) were performed using Minitab 16 software to define the operative conditions for the experimental design. The experimental results confirmed that the optimal conditions to obtain the higher yield of the most abundant metabolites include high solvent:plant ratio (12 *v*/*w*) and mild heating (50 °C) for 30 min. These conditions were selected to prepare the extracts that were tested in biological, pharmacological, and toxicological assays after being chemically characterized. Furthermore, details about the RSM are fully described in Supplementary material. The water extract of grape pomace was analyzed for the determination of the levels of selected phenols and flavonoids through quantitative HPLC-DAD-MS analysis. The list of compounds analyzed, the wavelengths, and the *m*/*z* ratio for their determination are listed in Table 2. Quantification was done through seven-point calibration curves with linearity coefficients (R2) > 0.999 in the concentration range of 2–140 µg/mL. The limits of detection were lower than 1 µg/mL for all the assayed analytes. The area under the curve from HPLC chromatograms was used to quantify the analyte concentrations in the extract. The chromatogram of the nine compounds assayed in the extract is reported in Figure 1: gallic acid (peak #1); caftaric acid (peak #2); catechin (peak # 3); chlorogenic acid (peak #4); epicatechin (peak #5); caffeic acid (peak #6); syringic acid (peak #7); coumaric acid (peak #8); ferulic acid (peak # 9). Catechin was the prominent compound (9.86 µg/mL); epicatechin, gallic acid, and caftaric acid were present at concentrations ranging from 2.33 to 4.16 µg/mL, whereas the concentrations of the other phenolic compounds were sensitively lower (0.49–1.05 µg/mL). The phenolic profile of the grape pomace extract is consistent with literature. Indeed, catechin, caftaric acid, and gallic acid were reported to be among the main phytochemicals present in both grape and pomace [21,22,23,24]. Intriguingly, catechins, besides having well known antioxidant activities [25], have been described as effective in treating and preventing different neurodegenerative and neurological disorders, although the heterogeneity of the studies reported in literature makes the drawing of reliable conclusions difficult [26]. Additionally, different studies suggest the capability of these compounds to cross the blood—brain barrier [27,28,29], which makes the evaluation of neuroprotective effects of extracts containing significant amounts of catechins sensible, as in the case of grape pomace. Therefore, after the evaluation of potential eco-toxicity, the water extract of grape pomace was administered to the hypothalamic cell line Hypo E22, and bio-pharmacological assays were conducted in order to focus on the neuroprotective effects of this extract.

### 2.2. Eco-Toxicological Assays

**Figure 2:**
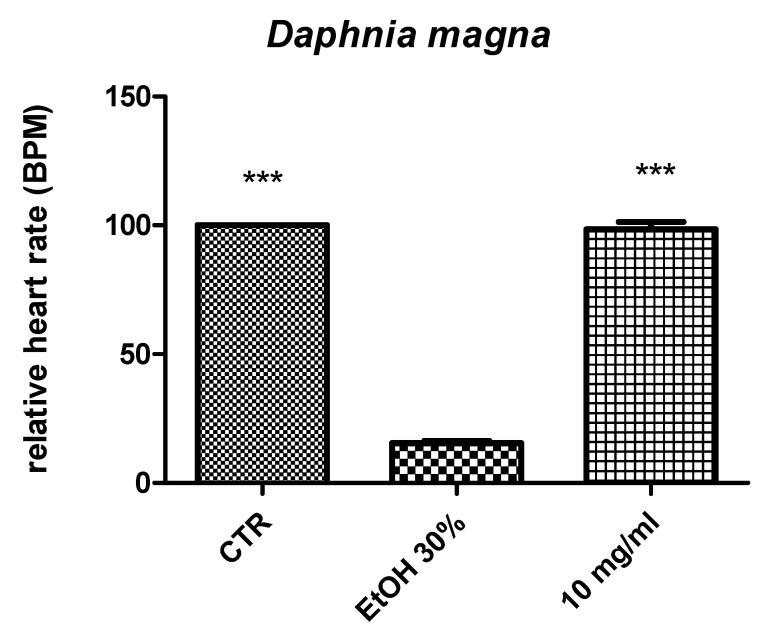
Effects induced by grape pomace water extract (10 mg/mL) on hearth rate in the *Daphnia magna* toxicity model. ANOVA, *p* < 0.0001; *** *p* < 0.001 vs. EtOH 30%, this last representing the positive control toxicity group.

The biological properties of the extract were initially assayed through the *Artemia salina* (brine shrimp) lethality assay with the aim of defining the biocompatibility of the extract in the concentration range of 0.1 to 20 mg/mL, as previously described [18]. As already observed for *Prunus mahaleb* [30], another edible plant, the water extract from grape pomace displayed a LC_50_ value >10 mg/mL. In addition, in the *Daphnia magna* toxicity test, the extract did not significantly alter the heart rate at the concentration of 10 mg/mL (Figure 2), thus scaling back the intrinsic toxicological properties of the extract. This is also consistent with the in silico eco-toxicological tests conducted on the present biological model. In this context, the putative LC_50_ values against *D. magna* for catechin, caftaric acid, and gallic acid were in the range of 3.64 to 144.19 mg/mL. However, considering the neuron susceptibility to oxidative/nitrosative stress [31] and the capability of antioxidants including phenolic compounds, to induce mild oxidative stress, [32], a concentration at least 10-fold lower compared to the LC_50_ value calculated in the brine shrimp assay was chosen for the subsequent experiments in hypothalamic HypoE22 cells.

### 2.3. Protective Effects in HypoE22 Cells

**Figure 3:**
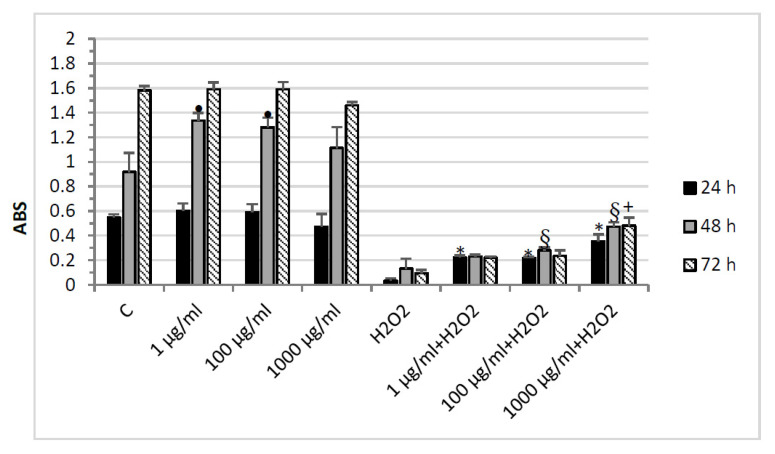
MTT assay of HypoE22 hypothalamic cell line exposed to different concentrations (1–1000 µg/mL) of water pomace extract for 24, 48, and 72 h. The data graph bars are the mean ± SD (*n* = 3). • *p* < 0.05 vs. 48 h C; * *p* < 0.05 vs. 24 h H_2_O_2_; § *p* < 0.05 vs. 48 h H_2_O_2_; + *p* < 0.05 vs. 72 h H_2_O_2._

Figure 3 shows the effect of the grape pomace extract on HypoE22 cell line viability. In basal conditions, there were no differences except for a slight increase in cell proliferation at 48 h for the two lower concentrations. However, when the cells were treated with H_2_O_2_, their viability was severely affected at all experimental times. The administration of the water extract increased the viability of the cells challenged with the oxidative stress. These results are in line with previous data about the protective effect exerted by pomace extract on cell viability [33], even though little is known about the potential neuroprotective effects of pomace extracts. On the other hand, the most represented phenols in the extract, catechin and epicatechin, proved able to protect neurons from oxidative stress [34]. The gallic acid not only prevented neuronal loss induced by an oxidative stimulus [35] but was effective also in an in vivo model in the region of the hypothalamus [36], in line with the finding in our experimental model.

**Figure 4:**
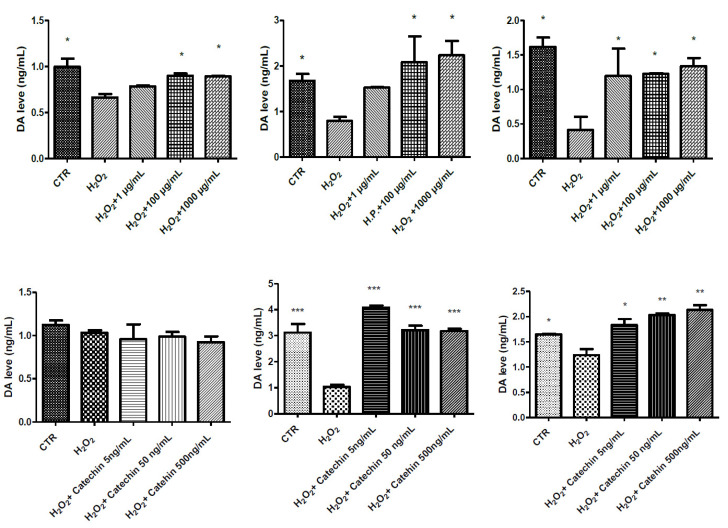
Inhibitory effects exerted by grape pomace water extract (1–1000 µg/mL) and catechin (5–500 ng/mL) on hydrogen peroxide (H_2_O_2_)-induced reduction of dopamine (DA) extracellular levels (ng/mL) in HypoE22 cells. The efficacy of the extract was evaluated at different time points: 24 h (**left**); 48 h (**middle**); 72 h (**right**). ANOVA, *p* < 0.0001; * *p* < 0.05, ** *p* < 0.01, *** *p* < 0.001 vs. respective hydrogen peroxide (H_2_O_2_) group.

In HypoE22 cells, the grape pomace extract was also tested to evaluate other parameters imbalanced by the burden of oxidative stress and induced by cell exposure to hydrogen peroxide. In this context, the extract blunted the hydrogen peroxide-induced dopamine degradation in the 72 h following extract treatment (Figure 4). This is consistent, albeit partially, with the pattern of phenolic compounds present in the extract and able to act as scavenging/reducing agents, thus contrasting the dopamine turnover induced by different pro-oxidant stimuli [37,38]. In this regard, the comparison with the effect exerted by the main phenolic compound in the extract, catechin, which showed efficacy only at 48 h and 72 h, seems to indicate that the protective effects on dopamine of the extract could also depend on other phenols.

**Figure 5:**
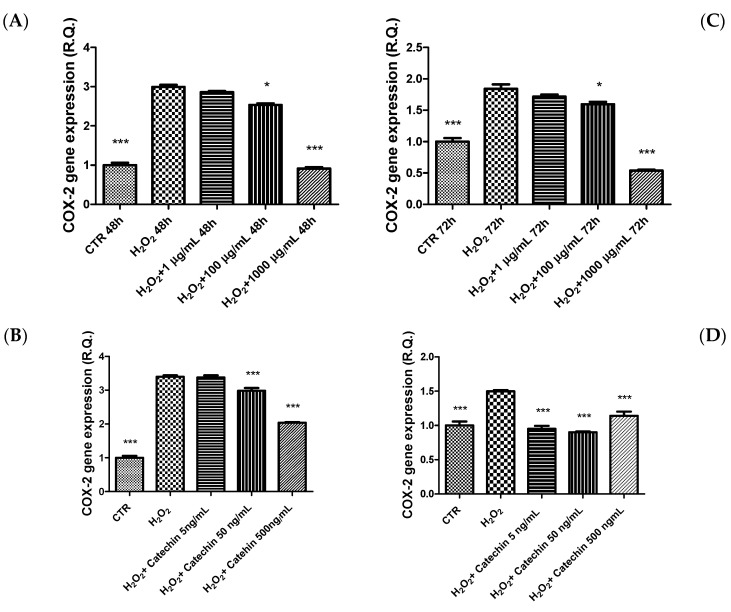
Inhibitory effects exerted by grape pomace water extract (1–1000 µg/mL) and catechin (5–500 ng/mL) on hydrogen peroxide (H_2_O_2_)-induced increase in cyclooxygenase-2 (COX-2) gene expression in HypoE22 cells. The efficacy of the extract was evaluated at different time points: 48 h (**A**,**B**); 72 h (**C**,**D**). ANOVA, *p* < 0.0001; * *p* < 0.05, *** *p* < 0.001 vs. respective hydrogen peroxide group (H_2_O_2_).

**Figure 6:**
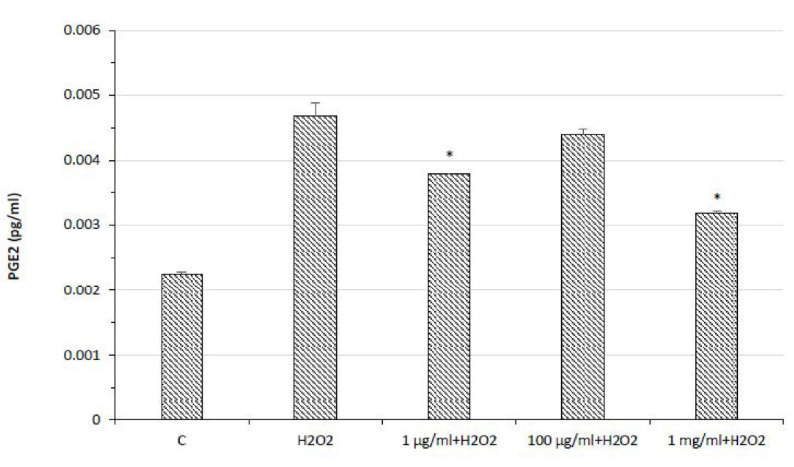
PGE_2_ production in HypoE22 hypothalamic cell line exposed to different concentrations (1–1000 µg/mL) of water pomace extract. The data graph bars are the mean ± SD (*n* = 3). * *p* < 0.05 vs. C.

In the same conditions, the extract was also effective in preventing the upregulation of cyclooxygenase-2 (COX-2) gene expression (Figure 5) and the release of prostaglandin E_2_ (Figure 6), the main COX-2-deriving prostanoid. Therefore, the extract showed remarkable anti-inflammatory effects, which are consistent, albeit partially, with the enzyme inhibition properties of the catechin against COX-2 [39]. These results also agree with the catechin submicromolar affinity toward COX-2 [40], whereas the actual catechin concentration in the present extract (9.86 µg/mL in 20 mg/mL of extract) further reinforces the hypothesis of this compound as the main phytochemical in the extract responsible of the COX-2 inhibition in HypoE22 cells. This hypothesis is confirmed, albeit in part, by the pattern of gene expression of COX-2 following HypoE22 exposure to catechin, whose inhibitory effect mirrored the one induced by the whole extract.

**Figure 7:**
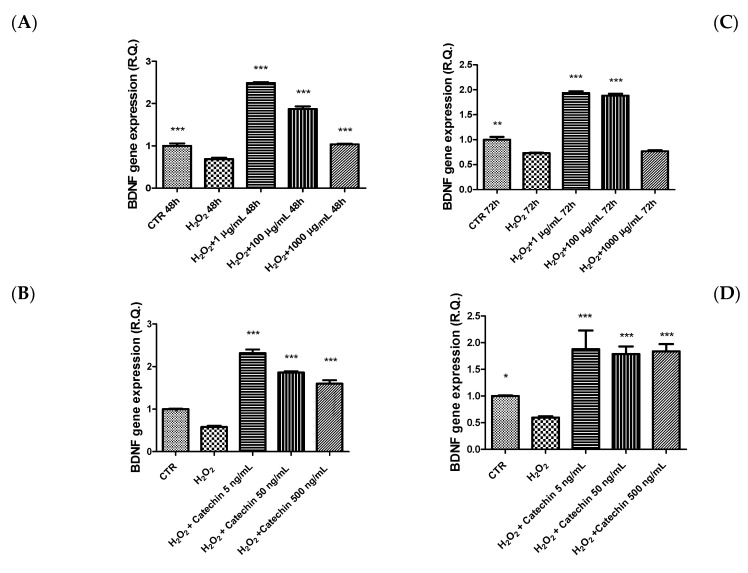
Inhibitory effects exerted by grape pomace water extract (1–1000 µg/mL) and catechin (5–500 ng/mL) on hydrogen peroxide (H_2_O_2_)-induced decrease in brain-derived neurotrophic factor (BDNF) gene expression in HypoE22 cells. The efficacy of the extract was evaluated at different time points: 48 h (**A**,**B**); 72 h (**C**,**D**). ANOVA, *p* < 0.0001; * *p* < 0.05, ** *p* < 0.01, *** *p* < 0.001 vs. respective hydrogen peroxide group (H_2_O_2_).

In parallel, the extract and the catechin alone were also able to blunt the hydrogen peroxide-induced reduction of the gene expression of BDNF (Figure 7), a neuropeptide deeply involved in neuron survival as well as in learning and memory [41]. The pro-homeostatic effects exerted by the extract on the tested biomarkers further strengthen the antioxidant properties of grape pomace-derived products [42]. In the hypothalamus, BDNF could act as an anorexigenic neuropeptide [43], whereas DA was reported as an appetite-stimulating factor [44]. Additionally, in this case, our data agree with literature suggesting the ability of catechin to mediate the inhibition of hydrogen peroxide-induced BDNF downregulation following extract treatment [45]. Finally, considering the tight relationships between hypothalamic neuromodulators and peripheral hormones, including leptin and insulin [46], our findings also support the potential use of grape pomace as an anti-obesity agent [47].

## 3. Materials and Methods

### 3.1. Grape Pomace Sample, Reagents, and Solutions

Pomace represented by only pressed grape fruits was kindly provided by a local farm in the village of Villamagna (GPS coordinates: 42°19′47.42″ N 14°14′12.8″ E), province of Chieti (Italy). The authentication of the plant material was done by Prof. Luigi Menghini. The pomace represents the residual waste product obtained after the preliminary fermentation subsequentially squeezed to recover the liquid phase. The resulting material appears as semisolid tablets that were manually disrupted and immediately lyophilized to remove the residual water. The dry pomace was stored in a sealed plastic bag under vacuum and kept in the dark at room temperature until extraction.

The plant material derives from the cultivation of local-native ecotypes of *Vitis vinifera variety* ‘Montepulciano’ used to produce a high quality red wine with Denomination of Controlled Origin (Villamagna DOC). According to the procedural guidelines, no less than 95% of the production is represented by the variety Montepulciano cultivated in selected geographical areas in the Abruzzo Region (Central Italy), limited to the municipalities of Villamagna, Vacri, and Bucchianico, in the province of Chieti (Italy). The soil exposure is south-east or south-west, and cultivation altitude is between 30 m and 180 m above the sea level with a low productive yield (less than 120 quintals per hectare) strongly characterized by organoleptic properties.

### 3.2. Grape Pomace Extracts Preparation Solutions

Dry pomace samples weighed using a Precisa XT220A balance (Micro Precision Calibration Inc., Grass Valley, CA, USA) were roughly homogenated with a T25 digital Ultra-Turrax tissue homogenizer (IKA, Staufen, Germany) in 50 mL Falcon tubes with bidistilled water (30 s at 10,000 g) in order to obtain uniform plant material size and to optimize the extraction. The homogenate was subjected to ultrasound-assisted extraction (UAE) in a Trans-sonic T460 ultrasonic bath (Elma, Singen, Germany). The operative conditions for the extraction were optimized through response surface methodology (RSM). A central composite, face-centered design at two levels with three factors full factorial design was defined to investigate the effects of selected variables (time of extraction, temperature, and solid:liquid ratio) for water UAE of grape pomace. The effects of independent variables were evaluated processing the extracts on HPLC-DAD-MS and applying a validated method for quantitative detection of eight common phenolic metabolites. The operative conditions as well as the extraction method (UAE) were selected on the basis of a previous study [30], while water was selected as the elective solvent for an ecofriendly, easy-to-use, homemade-oriented, and biocompatible extraction.

The experimental limits of independent variables applied to optimize the extraction through the RSM are summarized in Table 3.

### 3.3. High Performance Liquid Chromatography (HPLC) Analyses

The HPLC apparatus consisted of two PU-2080 PLUS chromatographic pumps, a DG-2080-54 line degasser, a mix-2080-32 mixer, UV, diode array (DAD) and detectors, a mass spectrometer (MS) detector (expression compact mass spectrometer (CMS), Advion, Ithaca, NY 14850, USA), an AS-2057 PLUS autosampler, and a CO-2060 PLUS column thermostat (all from Jasco, Tokyo, Japan). Integration was performed by ChromNAV2 Chromatography software. Before the injection in the HPLC apparatus, the grape pomace extract was centrifuged at 3500× *g* for 15 min, and the supernatant was diluted at 20 mg/mL.

### 3.4. HPLC-DAD-MS Determination of Phenolic Compounds

Grape pomace water extract was analyzed for phenol quantitative determination using a reversed-phase HPLC-DAD-MS in gradient elution mode, in agreement with literature data [48]. The separation was conducted within 36 min of the chromatographic run, starting from the following separation conditions: 95% water with 0.1% formic acid, 5% methanol with 0.1% formic acid, as already reported in literature [30]. The separation was performed on an Infinity lab Poroshell 120-SB reverse phase column (C18, 150 × 4.6 mm i.d., 2.7 µm) (Agilent, Santa Clara, CA, USA). Column temperature was set at 30 °C. Quantitative determination of phenolic compounds was performed via a DAD detector. The extract was also qualitatively analyzed with an MS detector in negative ion mode. MS signal identification was realized through comparison with a standard solution and MS spectra present in the MassBank Europe database (https://massbank.eu/MassBank/) (accessed on 31 July 2021).

### 3.5. High Performance Liquid Chromatography (HPLC) Determination of Dopamine (DA)

Extracellular DA levels were analyzed through an HPLC apparatus consisting of a Jasco (Tokyo, Japan) PU-2080 chromatographic pump and an ESA (Chelmsford, MA, USA) Coulochem III coulometric detector equipped with a microdialysis cell (ESA-5014b) porous graphite working electrode and a solid state palladium reference electrode. The analytical conditions for biogenic amine identification and quantification were selected according to a previous study [49]. Briefly, the analytical cell was set at −0.150 V for detector 1 and at +0.300 V for detector 2 with a range of 100 nA. The chromatograms were monitored at the analytical detector 2. Integration was performed by Jasco Borwin Chromatography software version 1.5. The chromatographic separation was performed by isocratic elution on a Phenomenex Kinetex reverse phase column (C18, 150 × 4.6 mm i.d., 2.6 µm). As regards the separation of DA, the mobile phase was (10:90, *v*/*v*) acetonitrile and 75 mM pH 3.00 phosphate buffer containing octanesulfonic acid 1.8 mM, EDTA 30 µM, and triethylamine 0.015% *v*/*v*. Flow rate was 0.6 mL/min, and the samples were manually injected through a 20 µL loop. Neurotransmitter peak was identified by comparison with the retention time of pure standard. Neurotransmitter concentrations in the samples were calculated by linear regression curve (y = bx + m) obtained with standard. The standard stock solution of DA at 2 mg/mL was prepared in bidistilled water containing 0.004% EDTA and 0.010% sodium bisulfite. The stock solutions were stored at 4 °C. Work solutions (1.25–20.00 ng/mL) were obtained daily by progressively diluting the stock solutions in the mobile phase.

### 3.6. Artemia Salina and Daphnia Magna Toxicity Tests

*Artemia salina* (brine shrimp) were cultured as previously described [30], and toxicity induced by the extract (0.1–20 mg/mL) was expressed in terms of LC_50_ values. *Daphnia magna* were cultured as previously described [50]. Briefly, non-pregnant *Daphnia magna* were maintained separately in 50 mL of extract solution (10 mg/mL) at room temperature throughout the experiment. The hearth rate was recorded through microscopy. *Daphnia magna* were placed individually onto a single cavity microscope slide in a 50 μL droplet of the tested extract, where the heartbeat rate was measured three times for 10 s. Each measurement was conducted in triplicate. The decrease in hearth rate was compared to both untreated and 30% ethanol-treated groups, working as negative and positive controls, respectively. In the case of extracts’ natural compounds, the toxicity on *Daphnia magna* was predicted through the Toxicity Estimation Software Tool (T.E.S.T.) as well, and the results were expressed as LC_50_ values.

### 3.7. Cell Culture and Treatment

The HypoE22 rat hypothalamus cell line was purchased from Cedarlane Corporation (Burlington, ON, Canada) and cultured in Dulbecco’s Modified Eagle Medium (DMEM) supplemented with 10% (*v*/*v*) heat-inactivated fetal bovine serum and penicillin–streptomycin (100 µg/mL) (all from EuroClone SpA Life-Sciences-Division, Milano, Italy). The cells were grown at 37 °C in a humidified atmosphere of 5% CO_2_. When indicated, the cells were treated with H_2_O_2_ (300 µM) for 3 h and with different concentrations of grape pomace extracts (1–1000 µg/mL).

### 3.8. MTT Assay

The cell viability was evaluated after 24, 48, and 72 h of culture by MTT (3-[4,5-dimethyl-thiazol-2-yl]-2,5-diphenyl tetrazolium bromide) growth assay (Sigma-Aldrich, St. Louis, MO, USA) based on the capability of viable cells to reduce MTT into a colored formazan product. The cells were seeded into 96-well plates at 5 × 10^3^ cells/well. At the established time points, the medium was replaced with a fresh one containing 0.5 mg/mL MTT, and the cells were incubated for 3 h at 37 °C. After a further incubation of the samples in DMSO for 30 min at 37 °C, the absorbance at 570 nm was measured using a Multiscan GO microplate spectrophotometer (Thermo Fisher Scientific, Waltman, MA, USA). The values obtained in the absence of cells were considered as background and subtracted from the optical density values of the samples. Three independent experiments were performed under the same experimental conditions.

### 3.9. PGE_2_ ELISA Assay

At 48 h, the cell supernatants were harvested, and the secretion of prostaglandin E2 (PGE_2_) in the culture media was evaluated by an ELISA kit (Enzo Life Sciences, Farmingdale, NY, USA), according to the manufacturer’s instructions. The optical density values were obtained by measuring the absorbance at 405 nm using a Multiscan GO microplate spectrophotometer (Thermo Fisher Scientific).

### 3.10. Gene Expression Analysis

Gene expressions of COX-2 and BDNF were measured as previously reported [48]. Briefly, after extraction through the TRI Reagent, total RNA was reverse transcribed using High Capacity cDNA Reverse Transcription Kit (Thermo Fischer Scientific). Gene expression was determined by quantitative real-time PCR using TaqMan probes obtained from Thermo Fischer Scientific. β-actin was used as the housekeeping gene. The analysis of data was conducted with the Sequence Detection System (SDS) software version 2.3 (Thermo Fischer Scientific). A detailed description of the experimental protocol is reported in a previous paper of ours [51].

## 4. Conclusions

The results of the present study highlight the valuable properties of the grape pomace, up to now considered as only waste material of the *V. vinifera* production chain. Intriguingly, the water extract revealed a good source of phenolic compounds, among which was catechin. The content of phenolics could explain, albeit partially, the observed antioxidant and neuroprotective effects in hypothalamic neurons, thus suggesting the use of grape pomace extracts as bioactive ingredients in food supplements to contrast the burden of oxidative stress occurring in chronic inflammatory conditions, including obesity. In this regard, the pro-homeostatic effects of grape pomace extract in restoring the basal hypothalamic dopamine and the BDNF levels following the exposure to oxidative stress stimulus support further investigations through in vivo paradigms for confirming the applicability as an anti-obesity agent. Overall, the results from the present study show the importance of grape pomace within local production chains as a high quality byproduct with promising applications as a health-promoting agent. Additionally, the use of an eco-friendly solvent, such as water in this case, strongly agrees with the principles of circular economy in terms of valorization of the byproducts of *V. vinifera* botanical chain and low environmental impact [52,53].

## Figures and Tables

**Table 1:** Design of the experimental matrix with resulting experimental data for detected metabolites.

Temperature (°C)	Time (min)	Solvent/Plant (*v*/*w*)	Gallic Acid	Caftaric Acid	Catechin	Chlorogenic Acid	Epicatechin	Caffeic Acid	Syringic Acid
20	5	4	3.066	3.326	1.522	1.043	1.532	0.354	0.789
80	5	4	3.473	3.349	1.708	1.052	1.601	0.38	0.769
20	55	4	3.452	3.368	2.096	1.0313	1.825	0.4	0.823
80	55	4	3.728	3.354	3.003	1.234	2.097	0.414	0.969
20	5	20	1.608	3.349	2.152	1.018	1.528	0.35	0.824
80	5	20	2.298	3.492	3.469	1.037	1.813	0.4	0.829
20	55	20	1.952	3.434	3.437	1.029	1.602	0.387	0.789
80	55	20	2.325	3.351	1.782	1.033	1.966	0.43	0.852
20	30	12	3.102	3.728	3.985	1.042	1.88	0.53	0.9
80	30	12	3.665	3.846	7.865	1.048	2.473	0.513	1.01
50	5	12	2.983	3.650	6.805	1.058	1.895	0.458	0.861
50	55	12	3.341	3.713	3.740	1.04	2.511	0.511	0.932
50	30	4	3.181	3.323	1.957	1.035	1.523	0.399	0.772
50	30	20	2.676	3.626	6.847	1.039	1.925	0.454	0.87
50	30	12	3.747	4.162	9.863	1.046	2.327	0.492	0.960

**Table 2:** Wavelengths of quantification, mass to charge (*m*/*z*) ratios and retention times related to the investigated phenolic compounds.

Standard	*m*/*z*	Wavelengths (nm)	Retention Time (min)
Gallic acid	169.1	254	7.937
Caftaric acid	311.2	254	10.483
Catechin	289.3	254	11.307
Chlorogenic acid	353.31	254	12.697
Epicatechin	289.3	254	14.653
Caffeic acid	179.16	254	16.313
Syringic acid	197.17	254	17.510
Coumaric acid	163.04	254	23.710
Ferulic acid	193.1	254	24.580

**Table 3:** Factors and relative levels applied to the experimental design.

Independent Variables	Levels		
	−1	0	1
Time (min)	5	30	55
Temperature (°C)	20	50	80
Solvent/plant material (mL/g)	4	12	20

## Data Availability

The data presented in this study are available on request from the corresponding author.

## Supplementary Materials

The following are available online, Figure S1: Effect of critical factors on phenolic and flavonoid extraction, Table S1: Estimated regression coefficient of the second order polynomial equation for response surface methodology analysis of secondary metabolite classes extraction.

## Sample Availability

Samples of the compounds and extract are available from the authors.
