# Supplementary material for: Phenolic Characterization and Neuroprotective Properties of Grape Pomace Extracts
Source: Molecules. 2021 Oct 14;26(20):6216. doi: 10.3390/molecules26206216 (PMC8540556; doi:10.3390/molecules26206216)
Supplement: Supplementary file 1 [file molecules-26-06216-s001.zip › molecules-1353297-supplementary.pdf]

*Response surface methodology (RSM)*

A set of central composite, face centered experiments of full factorial design at two levels, three factors with a total of 20 runs, in a single block, were applied to evaluate the curvature model. The independent variables were temperature, time and solvent/plant ratio, applied to an ultrasonic assisted extraction of dry pomace in water. The effects of each independent variables were extimated by quantitative HPLC of a set of phenolics Table S1 reports the estimated regression coefficients of the second order polynomial equations for RSM analysis of each metabolite detected in pomace water extract.

Regression coefficients for mean, linear, interaction and quadratic terms, were calculated respectively from the experimental results by the least squares method. Minitab 16 software was used. The ANOVA analysis was applied to evaluate the relevance of independent variables' influence and interactions ($p<0.05$). The adequacy of the model was predicted on the basis of the coefficient of determination (Rsq), the significance (p) and the lack of adjustment tests.

TABLE S1: Estimated regression coefficient of the second order polynomial equation for response surface methodology analysis of secondary metabolite classes extraction (uncoded).

| TERM | Estimated Regression Coefficients | P | Regression p-value | R squared | Lack of fit |
|---|---|---|---|---|---|
| **Gallic acid** | | | | | |
| Constant | 2.52718 | 0.023 | 0.001 | 88.54% | 0.574 |
| Temperature (°C) | 0.00299769 | 0.143 | | | |
| Time (min) | 0.0285591 | 0.000 | | | |
| Solvent/Plant (v/w) | 0.0748758 | 0.808 | | | |
| Temperature (°C)*Temperature (°C) | 4.56397E-05 | 0.298 | | | |
| Time (min)*Time (min) | -2.88679E-04 | 0.031 | | | |
| Solvent/Plant (v/w)*Solvent/Plant (v/w) | -0.00646757 | 0.574 | | | |
| Temperature (°C)*Time (min) | -7.46667E-05 | 0.633 | | | |
| Temperature (°C)*Solvent/Plant (v/w) | 0.000197917 | 0.734 | | | |
| Time (min)*Solvent/Plant (v/w) | -1.68750E-04 | 0.023 | | | |

| | | | | | |
|---|---|---|---|---|---|
| **Caftaric acid** | | | | | |
| Constant | 2.74663 | 0.722 | | 75.19% | 0.969 |
| Temperature (°C) | 0.00167682 | 0.918 | | | |
| Time (min) | 0.0133576 | 0.322 | | | |
| Solvent/Plant (v/w) | 0.124634 | 0.996 | | | |
| Temperature (°C)*Temperature (°C) | -5.72391E-07 | 0.301 | | | |
| Time (min)*Time (min) | -1.69624E-04 | 0.009 | | | |
| Solvent/Plant (v/w)*Solvent/Plant (v/w) | -0.00489086 | 0.577 | | | |
| Temperature (°C)*Time (min) | -4.38333E-05 | 0.913 | | | |
| Temperature (°C)*Solvent/Plant (v/w) | 2.65625E-05 | 0.826 | | | |
| Time (min)*Solvent/Plant (v/w) | -6.43750E-05 | 0.722 | | | |
| | | | | | |
| **Catechin** | | | | | |
| Constant | -7.03796 | 0.000 | 0.018 | 78.85% | 0.169 |
| Temperature (°C) | 0.159237 | 0.439 | | | |
| Time (min) | 0.198819 | 0.787 | | | |
| Solvent/Plant (v/w) | 1.16060 | 0.227 | | | |
| Temperature (°C)*Temperature (°C) | -0.00123588 | 0.334 | | | |
| Time (min)*Time (min) | -0.00282367 | 0.138 | | | |
| Solvent/Plant (v/w)*Solvent/Plant (v/w) | -0.0411765 | 0.037 | | | |
| Temperature (°C)*Time (min) | -3.75167E-04 | 0.671 | | | |
| Temperature (°C)*Solvent/Plant (v/w) | -7.45313E-04 | 0.786 | | | |
| Time (min)*Solvent/Plant (v/w) | -0.00141938 | 0.668 | | | |

| | | | | | |
|---|---|---|---|---|---|
| **Chlorogenic acid** | | | | | |
| Constant | 0.996257 | 0.038 | 0.054 | 72.55% | 0.000 |
| Temperature (°C) | 0.000429174 | 0.144 | | | |
| Time (min) | -5.80623E-04 | 0.039 | | | |
| Solvent/Plant (v/w) | 0.00576045 | 0.763 | | | |
| Temperature (°C)*Temperature (°C) | 6.59035E-06 | 0.616 | | | |
| Time (min)*Time (min) | 1.58901E-05 | 0.916 | | | |
| Solvent/Plant (v/w)*Solvent/Plant (v/w) | -3.23232E-05 | 0.075 | | | |
| Temperature (°C)*Time (min) | 2.97833E-05 | 0.062 | | | |
| Temperature (°C)*Solvent/Plant (v/w) | -9.82813E-05 | 0.099 | | | |
| Time (min)*Solvent/Plant (v/w) | -1.02063E-04 | 0.038 | | | |
| | | | | | |
| **Epicatechin** | | | | | |
| Constant | 0.855679 | 0.031 | 0.030 | 76.28% | 0.471 |
| Temperature (°C) | -0.00153196 | 0.027 | | | |
| Time (min) | 0.00284779 | 0.693 | | | |
| Solvent/Plant (v/w) | 0.163681 | 0.800 | | | |
| Temperature (°C)*Temperature (°C) | 3.47363E-05 | 0.641 | | | |
| Time (min)*Time (min) | 9.24202E-05 | 0.006 | | | |
| Solvent/Plant (v/w)*Solvent/Plant (v/w) | -0.00658183 | 0.628 | | | |
| Temperature (°C)*Time (min) | 4.70000E-05 | 0.597 | | | |
| Temperature (°C)*Solvent/Plant (v/w) | 0.000160417 | 0.343 | | | |
| Time (min)*Solvent/Plant (v/w) | -3.51250E-04 | 0.031 | | | |

| | | | | | |
|---|---|---|---|---|---|
| **Caffeic acid** | | | | | |
| Constant | 0.254769 | 0.336 | 0.018 | 78.98% | 0.642 |
| Temperature (°C) | -0.00116945 | 0.112 | | | |
| Time (min) | 0.00346747 | 0.534 | | | |
| Solvent/Plant (v/w) | 0.0309590 | 0.599 | | | |
| Temperature (°C)*Temperature (°C) | 1.31987E-05 | 0.278 | | | |
| Time (min)*Time (min) | -4.01939E-05 | 0.004 | | | |
| Solvent/Plant (v/w)*Solvent/Plant (v/w) | -0.00129877 | 0.857 | | | |
| Temperature (°C)*Time (min) | -3.16667E-06 | 0.617 | | | |
| Temperature (°C)*Solvent/Plant (v/w) | 2.76042E-05 | 0.902 | | | |
| Time (min)*Solvent/Plant (v/w) | -8.12500E-06 | 0.336 | | | |
| | | | | | |
| **Syringic acid** | | | | | |
| Constant | 0.656014 | 0.056 | 0.018 | 79.10% | 0.641 |
| Temperature (°C) | -0.00382217 | 0.064 | | | |
| Time (min) | 0.00324294 | 0.772 | | | |
| Solvent/Plant (v/w) | 0.0431347 | 0.202 | | | |
| Temperature (°C)*Temperature (°C) | 4.07800E-05 | 0.436 | | | |
| Time (min)*Time (min) | -3.48768E-05 | 0.005 | | | |
| Solvent/Plant (v/w)*Solvent/Plant (v/w) | -0.00152028 | 0.106 | | | |
| Temperature (°C)*Time (min) | 3.73333E-05 | 0.655 | | | |
| Temperature (°C)*Solvent/Plant (v/w) | -3.02083E-05 | 0.080 | | | |
| Time (min)*Solvent/Plant (v/w) | -1.53750E-04 | 0.056 | | | |

The ANOVA analysis of the model for the phenols metabolites extractions from grape shows that the models is significant ($p<0.05$) according to R squared and p- values for gallic acid, syringic acid, caffeic acid, benzoic acid catechin and epicatechin. The missing significance for the lack of fit in the model support the applicability of the model in prediction of selected metabolites recovery. The model highlights a suitability for prediction of catechin recovery (78.85% and P=0.169 as R Sq and Lack of fit. respectively), being catechin the most aboundant metabolite and the quadratic solvent-plant ratio a critical factor.

To visualize the relationship between the response and experimental levels of the independent variables for each detected metabolite, the contour plots were constructed according to the quadratic polynomial model equations and reported in Figures S1 A-G.

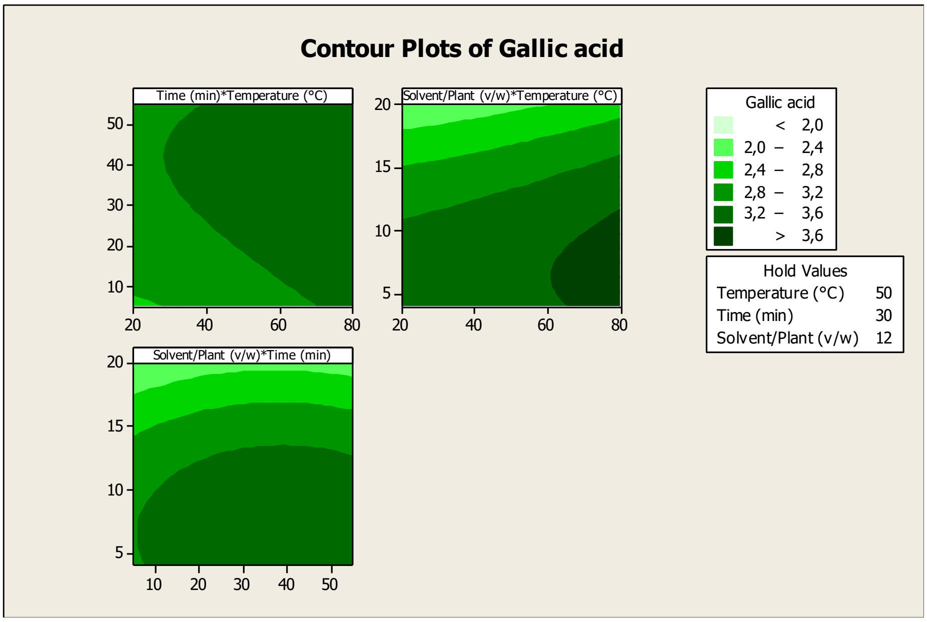


A

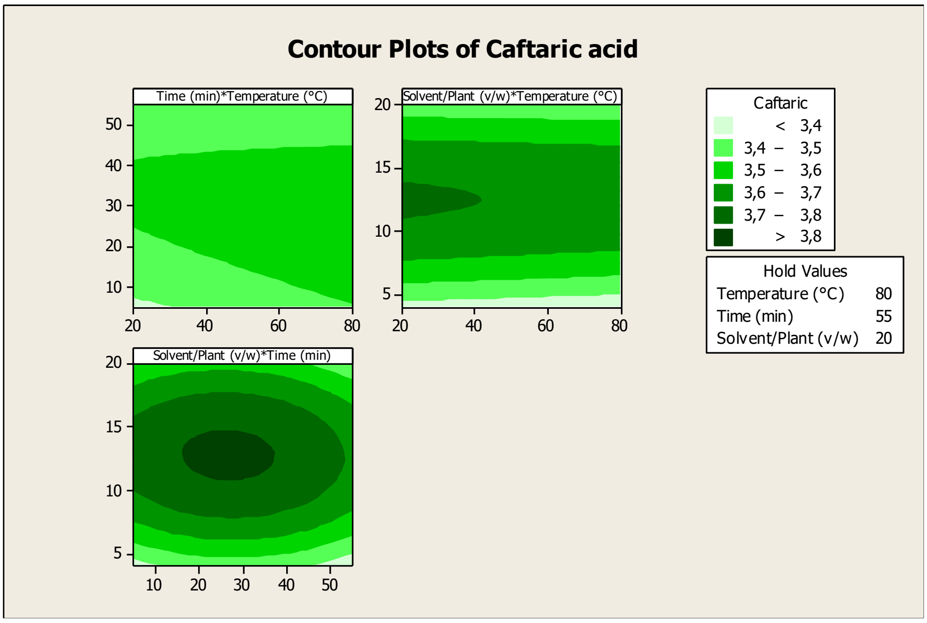
Contour Plots of Caftaric acid
Time (min)*Temperature (°C)
Solvent/Plant (v/w)*Temperature (°C)
Solvent/Plant (v/w)*Time (min)
Caftaric
< 3,4
3,4 – 3,5
3,5 – 3,6
3,6 – 3,7
3,7 – 3,8
> 3,8
Hold Values
Temperature (°C) 80
Time (min) 55
Solvent/Plant (v/w) 20

B

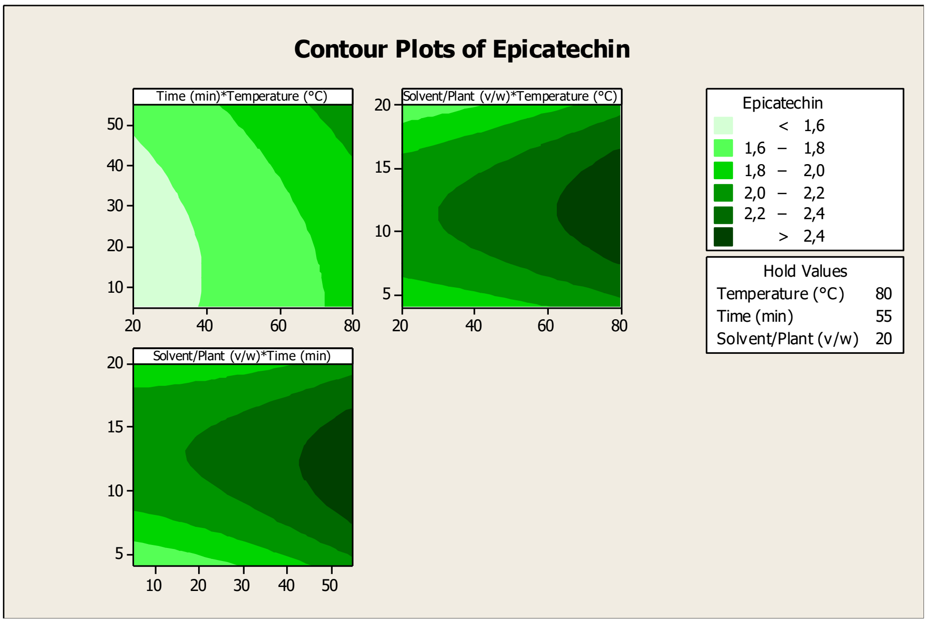
Contour Plots of Epicatechin
Time (min)*Temperature (°C)
Solvent/Plant (v/w)*Temperature (°C)
Solvent/Plant (v/w)*Time (min)
Epicatechin
< 1,6
1,6 – 1,8
1,8 – 2,0
2,0 – 2,2
2,2 – 2,4
> 2,4
Hold Values
Temperature (°C) 80
Time (min) 55
Solvent/Plant (v/w) 20

C

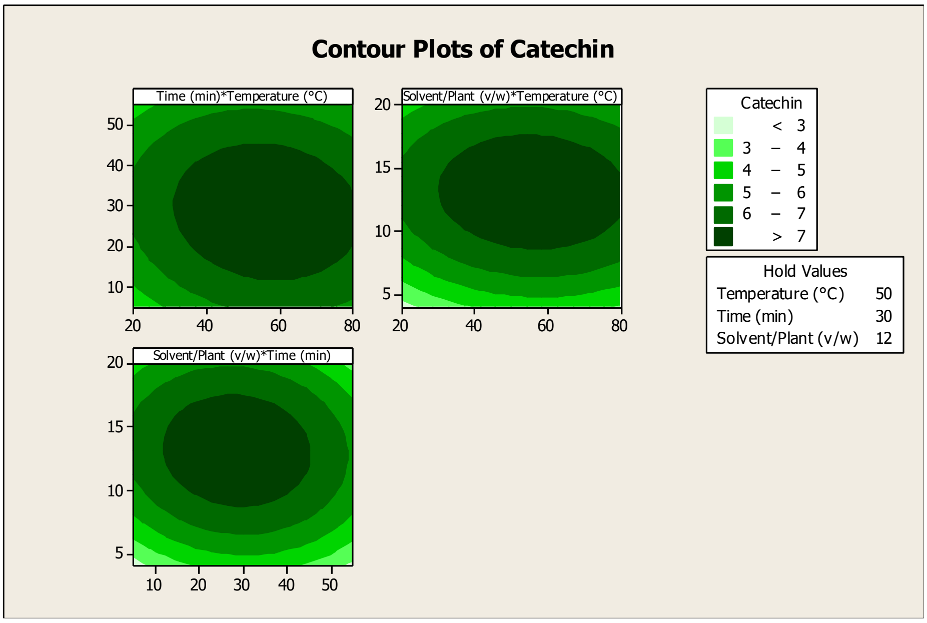
Contour Plots of Catechin
Time (min)*Temperature (°C)
Solvent/Plant (v/w)*Temperature (°C)
Solvent/Plant (v/w)*Time (min)
Catechin
< 3
3 – 4
4 – 5
5 – 6
6 – 7
> 7
Hold Values
Temperature (°C) 50
Time (min) 30
Solvent/Plant (v/w) 12

D

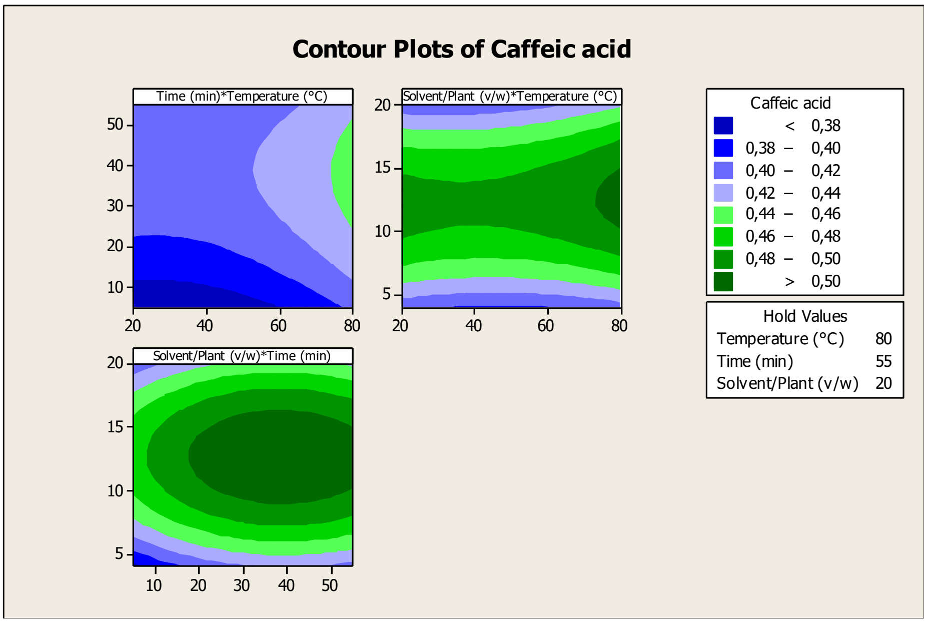
Contour Plots of Caffeic acid
Time (min)*Temperature (°C)
Solvent/Plant (v/w)*Temperature (°C)
Solvent/Plant (v/w)*Time (min)
Caffeic acid
< 0,38
0,38 – 0,40
0,40 – 0,42
0,42 – 0,44
0,44 – 0,46
0,46 – 0,48
0,48 – 0,50
> 0,50
Hold Values
Temperature (°C) 80
Time (min) 55
Solvent/Plant (v/w) 20

E

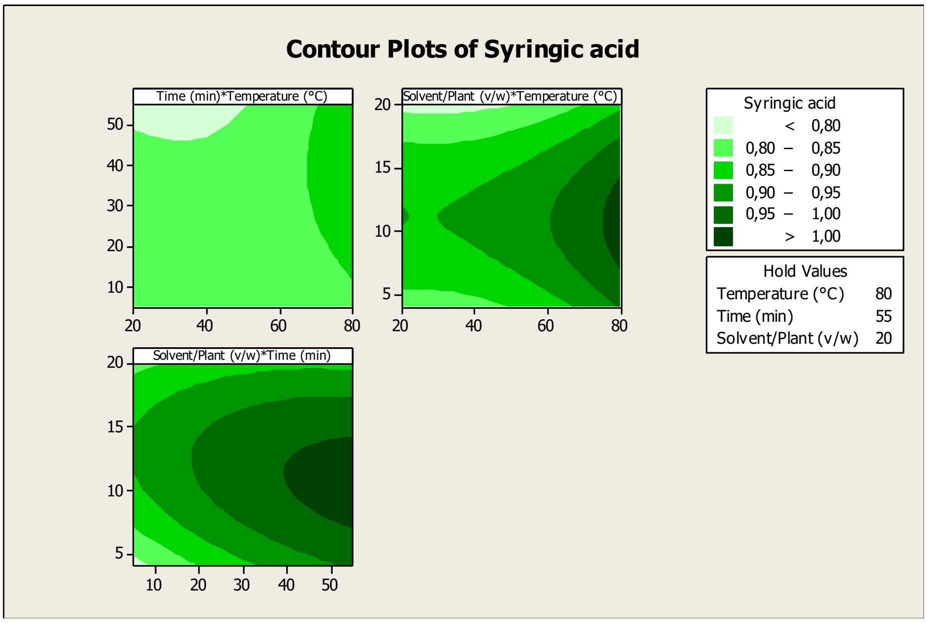


F

Figure S1. Effect of critical factors on phenolic and flavonid extraction (A-F): contour graphs of plant/solvent interaction with other variables at central point level showed the optimal conditions for catechin extraction (30 minutes, 12 Solvent/Plant ratio, temperature 50°C)
